# The feasibility study of transfer arc plasma pyrolysis system for petrochemical wastes: Hydrogen production from Antar, OTD, and Tar

**DOI:** 10.1016/j.heliyon.2024.e41451

**Published:** 2024-12-24

**Authors:** Amir Hossein Kheyriyeh, Farzaneh Ostovarpour, Mohammadreza Khani, Mohammad Sadegh Abbassi Shanbehbazari, Babak Shokri

**Affiliations:** a Laser and Plasma Research Institute, Shahid Beheshti, University, G.C., Evin, 19839-63113, Tehran, Iran; b Department of Physics, Shahid Beheshti University, G.C. Evin, 19839-63113, Tehran, Iran

**Keywords:** Plasma pyrolysis, Petrochemical waste, Thermal plasma, Hydrogen production

## Abstract

One of the best and most advanced methods for disposal of urban, hospital, industrial, and other hazardous waste is to convert waste into combustible gases in reactors based on plasma arc technology. Also used for renewable energy generation, this technology involves thermal treatment without a combustion process; therefore, the waste is completely decomposed into simple molecules in a near vacuum environment almost devoid of Oxygen at elevated temperatures. The present research uses a thermal transferred arc plasma reactor to conduct a feasibility study on the pyrolysis of three types of wastes: Antar, Orthotoluenediamine (OTD), and Tar. To this end, three experiments were conducted, and the effective parameters involved in preparing and producing common gaseous raw materials and products, such as hydrogen, and their production amounts were examined. The outcome demonstrates that there are no remnants of spent catalyst waste following the plasma pyrolysis process, confirming the feasibility of complete conversion. The findings of this study revealed that plasma waste reactors could generate recycled energy in the form of renewable fuel free of toxic gases and vapors released from conventional waste incinerators and create negligible amounts of environmental pollutants.

## 1 Introduction

Plasma, the electrified fourth state of matter, is no longer confined to theoretical physics; it has become a dynamic cornerstone of modern science and technology. Over recent years, scientists and engineers have extensively explored its diverse applications [[1], [2], [3], [4], [5], [6], [7], [8], [9]], from revolutionizing energy production to pioneering breakthroughs in materials science and healthcare. The thermal plasma is an active environment comprising electrons, ions, and neutral atoms. Once the ionized species are recombined with the detached electrons, a large amount of energy will be released through ultraviolet radiation. Consequently, plasma will be transformed into a fully active environment that triggers some specific chemical reactions, hence the decomposition of many homogeneous and heterogeneous chemical compounds. In recent decades, thermal plasma has gained broad applications in waste disposal, particularly in the disposal of special wastes [3,[10], [11], [12], [13], [14]]. Many plasma facilities have been constructed worldwide, especially in advanced industrialized countries where thermal plasma is efficiently employed to dispose of standard and special wastes [[15], [16], [17], [18], [19], [20], [21], [22]]. Each of these facilities operates pursuing a particular objective, either economic [[23], [24], [25]] or environmental, or both, depending on the type of waste.

During the plasma interaction with waste, the waste is placed inside the plasma environment and directly exposed to active species at high temperatures. As a result, it undergoes various chemical reactions. The type of waste, its chemical compounds, and the plasma parameters, such as the amount and type of active species and temperature, are the key factors determining the nature of such reactions. In general, waste undergoes three main types of chemical reactions, which play a fundamental role in destroying unique waste compounds and ensuring other remarkable benefits of this method. The first reaction is pyrolysis [26,27], which can be defined as the chemical decomposition in an environment devoid of Oxygen at considerably high temperatures. In other words, when materials are exposed to an environment at temperatures higher than about 10,000 K in the absence of Oxygen, they undergo thermochemical decomposition, which, as an extremely powerful chemical reaction, converts all molecular and chemical bonds into their constituent elements [28]. The second reaction is gasification, which occurs when the specific conditions of the thermal plasma affect the organic materials in the waste and change them [29,30]. The output product of this chemical reaction is a combustible gas called syngas, mainly composed of carbon monoxide and hydrogen [31,32]. Syngas has a high heat value and, consequently, a high energy production rate; hence, it is commonly used to generate electricity in large and advanced facilities [[33], [34], [35]]. The gasification process is carried out in the temperature range of 4000–5000 °C in the core of the plasma reactor. This temperature range makes it feasible to speed up the reactions in the reactor and obtain valuable products. The third stage is vitrification. In this stage, inorganic waste materials such as metals, glass, and soil are transformed into vitrified slag using thermal plasma. These materials are of high economic value and are commonly used as raw materials in the road and construction industries [[36], [37], [38], [39], [40], [41], [42], [43], [44], [45]].

Thermal plasma is the only method among other waste disposal methods that can efficiently dispose of unique waste compounds in most cases. The hazardous waste components are usually so strong that other methods typically fail to break them down, and only plasma pyrolysis proved to be able to neutralize and disintegrate these compounds. Owing to their beneficial functions, plasma facilities have been set up with high energy efficiency and relatively small sizes, thus making it possible to transfer the facilities to waste production or collection sites. In this case, there is no need to transport special wastes that require special facilities with high costs [[46], [47], [48], [49], [50], [51], [52]].

In a study, Fung Fuh Wong et al. (2006) employed thermal plasma and gasification methods to economically reduce and recover Nickel oxide in the spent nickel-based catalyst while generating syngas from the partial oxidation of organic Tar [53]. Later, Marafi et al. (2016) presented a list of 8–10 petroleum refining processes that produce hazardous catalytic wastes [54]. Burkhard et al. recycled some spent catalysts as metal-containing waste through various plasma processes [55]. In a research paper published in 2020, Karimi et al. examined the conversion of the spent petrochemical catalyst wastes into valuable by-products using transferred thermal plasma technology [3].

Kuo-Chen Chiang et al. (2011) further advanced thermal plasma processing to recover the spent alumina-supported platinum catalyst (Pt/Al2O3) and reduce the valuable Platinum oxide. Their findings confirmed the decomposition of the organic Tar on the surface of the spent catalysts and its subsequent conversion into syngas, i.e., the reducing agents in the chemical reactions [56].

Aghayee et al. conducted an empirical study affirming the practicality of the transferred thermal plasma technology for spent caustic disposal. Their investigation employed gas chromatography (GC) analysis, revealing the complete conversion of spent caustic, with hydrogen identified as the primary product of the process [1]. Fasihi's research explores plasma pyrolysis as an environmentally friendly and suitable method, resulting in the generation of value-added products. The uniqueness of the study lies in the formulation of a method for the disposal of spoiled milk. The study achieved a maximum hydrogen yield of 60.396 % [2].

A thermal plasma torch serves as an example of a thermal plasma generator capable of providing suitable conditions for converting various types of waste owing to its high temperature. The shape and material of the electrodes, as the primary components in the plasma torch, vary depending on their industrial applications. Historically, Graphite was predominantly used in electrode manufacturing, and cooling methods were not usually employed in the manufacturing process. For this reason, its corrosion rate was relatively high during the operation, increasing the need for their replacement after a short period. In addition, pollution in the operating environment is an inevitable result of such corrosion, which is undesirable in some industrial applications, especially in surface engineering [48,[57], [58], [59]]. RF torches have been commonly applied in the past years; however, due to the advancements in the design of DC torches, RF torches have seen reduced usage. The current study also implemented this technique in the plasma waste disposal systems due to the significant advantages of DC torches and available facilities and equipment [60,61]. The main objective these researchers pursued was to discover the hidden capacities of petrochemical wastes to produce Hydrogen as a clean fuel and fuel of the future. It should be noted that few scientific sources have delved into plasma disposal methods, and the only solution currently found to this problem is landfilling [62,63]. The authors hope that the results of this research will broaden our horizon concerning the applications of this technology for valuable compound production. In this project, for the first time, the plasma pyrolysis method was utilized to examine and analyze three categories of petrochemical waste: Antar, OTD, and Tar.

## 2 Materials and methods

Gasification and pyrolysis are non-combustible thermal processes where high temperatures in an Oxygen-free or partial oxidation environment contribute to breaking down the inlet waste materials into simple molecules such as CH_4_, H_2_, CO_2_, CO, etc., and also transforming them into ash and slag. Given that CO_2_ in the syngas is of low value, its amount should be kept as low as possible to enhance the system's efficiency. The CO/CO_2_ ratio is considered a determining parameter in controlling the gasification process, and it increases upon increasing the temperature [64,65].

Tar, characterized by its dark, dense, and viscous nature, originates from diverse organic sources such as wood, coal, peat, and petroleum. Within the petrochemical realm, coal tar and petroleum tar are frequently encountered. Coal tar is a byproduct of coal carbonization, while petroleum tar is derived from the distillation process of crude oil. The term 'Tar petrochemical waste' specifically denotes waste materials encompassing tar compounds generated as byproducts of petrochemical processes. The makeup of Tar varies contingent on its origin, generally constituting an intricate blend of organic compounds encompassing aromatic hydrocarbons, phenols, and heterocyclic compounds. Tar has historical applications in waterproofing, wood preservation, and as a construction sealant. However, using Tar and its derivatives, particularly coal tar, raises environmental concerns due to the potential presence of hazardous substances. Improper handling and disposal can pose environmental challenges. Certain tar compounds, notably those containing polycyclic aromatic hydrocarbons (PAHs), are linked to health risks, given PAHs' classification as known carcinogens [66]. TDI (toluene diisocyanate) is synthesized using a chemical process involving toluene, a petroleum hydrocarbon. The production of TDI commonly entails the reaction of toluene with phosgene, followed by treatment with an appropriate amine compound. In producing TDI (toluene diisocyanate), a byproduct called Orthotoluenediamine (OTD) is generated. OTD is a diamine compound that is derived from toluene. OTD is categorized as a waste product within the scope of isocyanate production. In this context, TDI and OTD are designated waste byproducts due to their status as secondary compounds in the production process. TDI is the intended product, whereas OTD is an incidental byproduct co-produced with TDI. The classification of TDI and OTD as waste underscores the significance of implementing efficient waste management protocols within the chemical industry. Diligent management and proper disposal of these byproducts are imperative to mitigate environmental consequences and align with regulatory requirements. Antar commonly denotes a particular category of waste material or input substance obtained from diverse industrial procedures, frequently comprising a blend of organic and inorganic compounds. It might arise due to chemical production, petroleum processing, or alternative industrial operations. The makeup of Antar can fluctuate depending on its origin and the methodologies employed, yet it frequently encompasses elements such as hydrocarbons, aromatic compounds, and additional organic and inorganic components. Antar may be scrutinized in scientific investigations for its prospective applications in waste handling, recycling, or conversion into beneficial commodities via techniques such as pyrolysis, gasification, or chemical alteration. Antar is a descriptor that characterizes a sophisticated blend of discarded materials commonly sourced from industrial operations such as petrochemical production, organic synthesis, or chemical manufacturing. Usually, it comprises diverse organic substances, encompassing hydrocarbons, aromatic compounds, and other organic contaminants, alongside inorganic elements like metals, salts, and mineral remnants. Due to its varied composition and potential environmental implications, managing and disposing of Antar poses challenges. However, it also provides opportunities for recycling, recovery, and conversion into valuable resources. Scientists and industrial sectors have been investigating diverse technologies and methodologies to efficiently process and utilize Antar. These include thermal techniques such as pyrolysis and gasification, chemical conversion approaches, and biological treatments. Comprehending the composition and dynamics of Antar is essential for devising sustainable waste management tactics and alleviating environmental hazards linked to its disposal. Investigations in this domain strive to enhance waste treatment procedures, reduce environmental contamination, and exploit the potential of Antar as a valuable asset for sustainable manufacturing and energy generation.

This research used a thermal transferred arc plasma reactor [2,3]. The temperature of the transferred arc plasma exceeds that of the non-transferred one. In this reactor, the waste is directly processed by the arc. The fundamental distinction between transfer arc and non-transfer arc plasma pyrolysis systems resides in the method by which the electric arc is initiated and sustained within the system. In transfer arc systems, the initiation and propagation of an electric arc occur as it transitions from one electrode to another, facilitated by the material undergoing processing, which is the waste feedstock. The arc originates at one electrode and is conveyed to the opposite electrode through the conductive characteristics of the material, typically as the waste traverses the plasma zone. Implementation of moving electrodes or a rotating drum is common in transfer arc systems to enhance the transfer of the electric arc along the material. In non-transfer arc systems, the electric arc is confined to a defined region, and the material undergoing processing does not actively facilitate the arc transfer. The arc is generated and maintained within a localized area, while the material is subjected to high-temperature plasma without directly contributing to the electrical discharge [52,67,68].

**Fig. 1:**
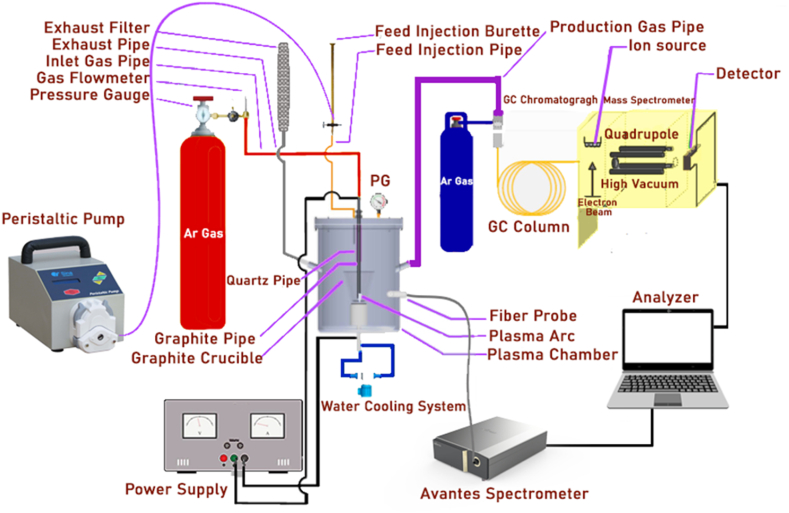
Schematic diagram of transfer plasma pyrolysis experimental setup.

In all experiments, the inlet gas flow was set at 10 SLM. Fig. 1 shows the schematic of the stainless-steel chamber of the thermal arc plasma reactor. It should be noted that this chamber is not damaged temperature-wise, and the only possible damage would be the sputtering phenomenon. Of note, the effect of this phenomenon diminishes until it reaches zero when the anode is placed inside the crucible (cathode). The mean temperature of plasma was measured according to our previously published paper [1] with the optical emission spectroscopy method in 180 A without a stainless steel chamber. It was near 16,258.27 K, and the reactor wall temperature was near the ambient temperature (less than 40 °C).

To investigate the experiments further, the collected gas products in all the experiments were injected into a gas chromatography (GC) device. Equipped with specific columns and detectors, this device can determine the type of produced products in molar percentage. In this study, a chromatography device called Agilent Technologies-7890A System was employed.

Following completing the above steps and closing the chamber lid, Argon gas is first injected with a flow of 30 SLM and for 3 min until the volume of the reactor chamber, which is about 50 L, is emptied of air, and there is no Oxygen in the environment. Since the reactor is large and has non-isolated inlets and outlets, remaining a small amount of air inside it is still inevitable. Upon turning on the power source and then forming the plasma, the plasma was kept on for 1 min, and during this time, the Argon gas flow was reduced to 10 SLM to make the formed plasma a little stable and prevent shutting down during the operation. Then, feed injection begins at a constant rate while simultaneously taking the injection time. The feed was injected with Peristaltic pumps at a constant flow of 10 cc/min for 2 min on the graphite crucible where the plasma is exactly produced. In order to make the amount of injection visible, a feed injection burette was used. After injecting half of the feed (1 min), sampling begins and continues until the end of the sample injection. After completing the sampling and separating the balloon from the silicone hose, the system will be shut down. Due to the very high temperature of the plasma flame that is created inside the graphite crucible, we see the corrosion of the graphite electrodes, which leads to a change in the distance between the electrodes and, as a result, the characteristics of the plasma. Due to the fact that it was not possible to keep the electrode distance constant in long-term operations, we considered the total test time to be 3 min (1 min for flame stability and 2 min for feed injection). In longer functions, reaching a quasi-steady state was impossible due to the aforementioned, and the test was associated with problems. Also, during this period, due to the constant argon gas flow and the exhaust system, hot gas flow adjacent to the flame was transferred to the outside of the reactor. Also, most of the flame radiation was absorbed by the graphite crucible, and as a result, the steel wall, which plays the role of the reactor wall, eventually reaches about 40 °C. To prevent interference between the previous and next experiments, the chamber lid will be opened to return the initial conditions of the test to the last state.

## 3 Results

The tests in this study were conducted on three types of waste: Antar, OTD, and Tar. The wastes under investigation will be analyzed in the following to evaluate the performance of plasma during the disposal process. A sample was extracted from the outlet gases using balloons evacuated in advance by a rotary vacuum pump to prevent variations in the results of the conducted tests. As already mentioned, a GC device was used to analyze the sample. The result obtained from the gas analysis was quite interesting; a significant proportion of the feed amount was converted into Hydrogen gas and the rest into Carbon monoxide, Carbon dioxide, and Hydrocarbons. During the experiments, attempts were made to keep constant the values of the amount of injected feed (20 cc), reaction time, total energy given to the plasma and consequently to the feed, inlet gas flow, and gap distance between the anode and cathode. However, in the case of any variation in the mentioned variables, the results were re-evaluated.

Table 1, Table 2, Table 3 show the composition and components of the waste materials used in this research. The components of Antar used in this study are Nitrobenzene, Aniline, Aniline Tar, Schiff base, Water, and other inorganics. The OTD waste comprises Ortho Toluene Diamine, Meta Toluene Diamine, Toluidine, and Water. Tar contains different molar percentages of Carbon, Hydrogen, Oxygen, Nitrogen, Chlorine, Pd, and Pt, among which Carbon has the largest share. Table 4 presents the physical characteristics of these three wastes (along with the units of quantities), where NA stands for Not Available. In this table, the heating values refer to the amount of energy obtained from burning a unit of mass, which is classified into two groups: low heating value (LHV) and high heating value (HHV) [69]. The former refers to a condition where water remains as a liquid in the combustion chamber at the end of the combustion process.

**Table 1:** Antar components.

Antar Components	Nitrobenzene	Aniline	Aniline Tar	Schiff base (%)	Water	Other inorganics
Average MW (−)	123	93	126	209	18	168
Mass flow (Kg/hr)	8.7	32	10	20	0.033	0.01
wt (%)	12.31	44.87	14.61	28.14	0.05	0.01

**Table 2:** OTD components.

OTD Components	Ortho Toluene Diamine	Meta Toluene Diamine	Toluidine	Water
Average MW (−)	122	122	107	18
Mass flow (Kg/hr)	159	0.003	0.44	0.011
wt (%)	99.71	–	0.28	0.01

**Table 3:** Tar components.

Tar Components	Carbon	Hydrogen	Oxygen	Nitrogen	Chlorine	Pd and Pt
Mol (%)	65	4	13	17	0.9	5.2 ppm

**Table 4:** Physical characteristics of Antar, OTD, and Tar wastes.

Waste	Antar	OTD	Tar
Op. Temp. (C°)	176	133	NA
Op. Press. (Bar)	4.5	3.2	NA
Average MW (−)	120	122	NA
Density (Kg/m^3^)	873	958	1060
Specific Heat (KJ/Kg k)	2.14	2.77	NA
Viscosity (mPa.S)	0.32	3.74	NA
Enthalpy (kJ/kg)	634	194	NA
Thermal Conductivity (W/mK)	0.12	0.17	NA
Max Flow (m^3^/hr)	1.4	0.18	NA
Max Flow (Kg/hr)	1193	176	1000
Normal Flow (m^3^/hr)	0.081	0.17	0.17
Normal Flow (Kg/hr)	71	160	160
Ash	NA	NA	0.1–0.6
LHV (Kcal/Kg)	NA	NA	6340

In contrast, the latter refers to a condition where the amount of energy obtained from burning a mass unit makes water exit the combustion chamber in the form of steam at the end of combustion. Other parameters, such as the humidity, amounts of combustible materials, and ash, can affect the heating value. In this research, only the LHV of Tar waste was available.

During waste plasma interaction, the waste undergoes direct exposure to high-temperature active species within the plasma environment, leading to various chemical reactions. The attributes of the waste, including its chemical composition, along with the plasma parameters, such as the abundance and type of active species, as well as temperature, play a critical role in defining the precise nature of these reactions [1,3]. As observed in Table 5, the current and applied voltage in all tests equals 180 A and 35 V, respectively. As a result, the power applied to the plasma is 6.3 KW. According to the results given in Table 5, Hydrogen is the main product of the plasma pyrolysis process, which accounts for 55.01 % of the products in the Antar waste disposal (0.555 SLM), 66.24 % (0.871 SLM) in the OTD waste disposal (the maximum value in this research), and 52.91 % (1.32 SLM) in the tar waste disposal (the third test). The percentages of the Carbon monoxide gas production in the three experiments are 36.37 % (0.367SLM), 17.87 % (0.235SLM), and 40.165 % (1.002SLM), respectively. The percentage of Carbon dioxide production in all tests was below 5 %, confirming that the combustion process did not occur. Based on the data of this research, it can be concluded that applying thermal plasma for waste disposal leads to the production of methane, ethanol, ethylene, propane, n-butane, and n-C4. The different results of each product are as follows:

**Table 5:** The measured data for the production of products.

	ANTAR	OTD	TAR
Voltage (V)	35	35	35
Current (I)	180	180	180
**H**_**2**_			
%	55.01	66.24	52.91
SLM	0.555	0.871	1.32
**CO**			
%	36.37	17.87	40.16
SLM	0.367	0.235	1.002
**CO**_**2**_			
%	3.07	2.13	3.41
SLM	0.031	0.028	0.085
**Methane**			
%	2.38	10.88	2.37
SLM	0.024	0.143	0.059
**Ethane**			
%	0.00	0.08	0.00
SLM	0.000	0.001	0.000
**Ethylene**			
%	1.39	0.99	0.20
SLM	0.014	0.013	0.005
**Propylene**			
%	0.10	0.02	0.00
SLM	0.001	0.000	0.000
**n-Butane**			
%	0.00	1.83	0.96
SLM	0.000	0.024	0.024
**n-C**_**4**_			
%	1.69	0.00	0.00
SLM	0.017	0.000	0.000
**Total Hydrocarbons**			
%	5.56	13.80	3.53
SLM	0.056	0.468	0.088
**H**_**2**_**/CO**	1.51	3.71	1.30
**CO**_**2**_ + **Methane**			
%	5.45	13.01	5.78
SLM	0.055	0.171	0.144
**H**_**2**_ + **CO**			
%	91.38	84.11	93.07
SLM	0.992	1.106	2.322
**CO/CO**_**2**_			
%	11.85	8.39	11.78
SLM	11.839	8.393	11.778

**Fig. 2:**
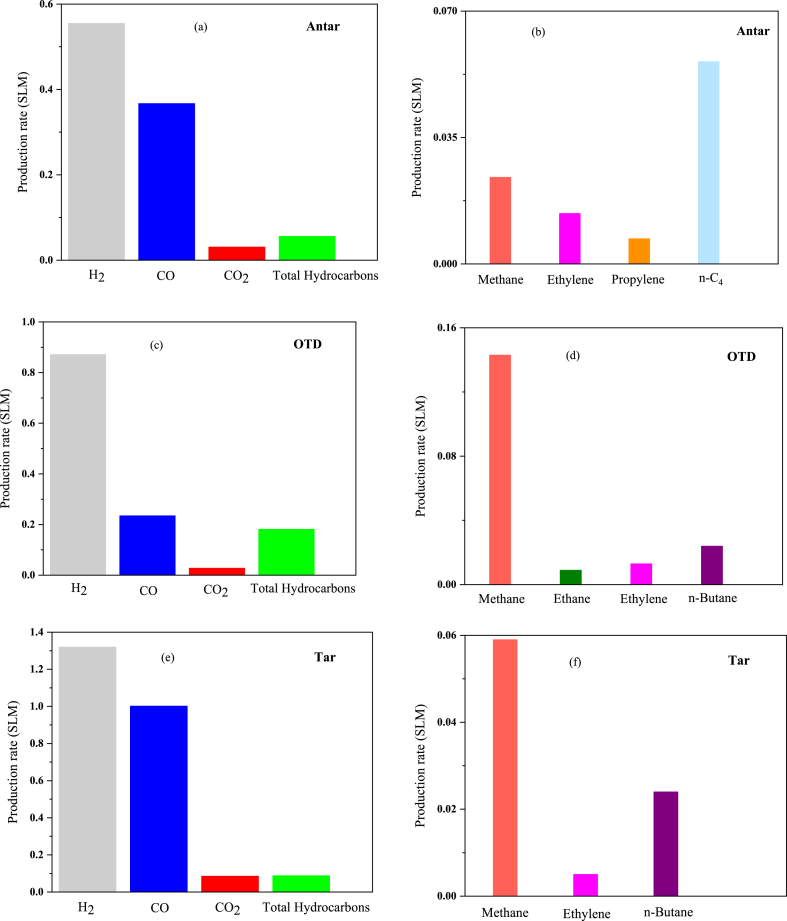
(a) – 2(f) Production rates at I = 180 A, V = 35 V for Antar, OTD, and Tar waste.

The percentage of Methane production ranges from 2.37 % (0.059SLM) to 10.88 % (0.143SLM). The rate of Ethane production in both Tar and Antar wastes is 0, while it is equal to 0.08 % (0.001SLM) in the OTD waste. The percentage of Ethylene production followed a decreasing trend, i.e., from 1.39 % (0.014SLM) to 0.20 % (0.005SLM). The production percentage of Propylene showed a slight change from 0 % to 0.1 % (0.001SLM). The rate of n-Butane production in the OTD waste increased from zero to 1.83 % (0.028SLM), while it decreased by 0.96 % (0.024SLM) in the Tar waste. The production percentage of n-C4 in the Antar waste is 1.69 % (0.017SLM), while it reached zero in other wastes. Fig. 2(a)–(f) present these results in SLM. Standard Liters per Minute (SLM) constitutes a foundational measure extensively employed across diverse disciplines, notably within gas dynamics and fluid mechanics. This metric quantifies the volume of gas, commonly air or an alternate gas, traversing a system amidst standard temperature and pressure conditions, frequently denoted as liters per minute (L/min). SLM assumes a pivotal role as a parameter of significance in myriad industrial and scientific applications.

**Fig. 3:**
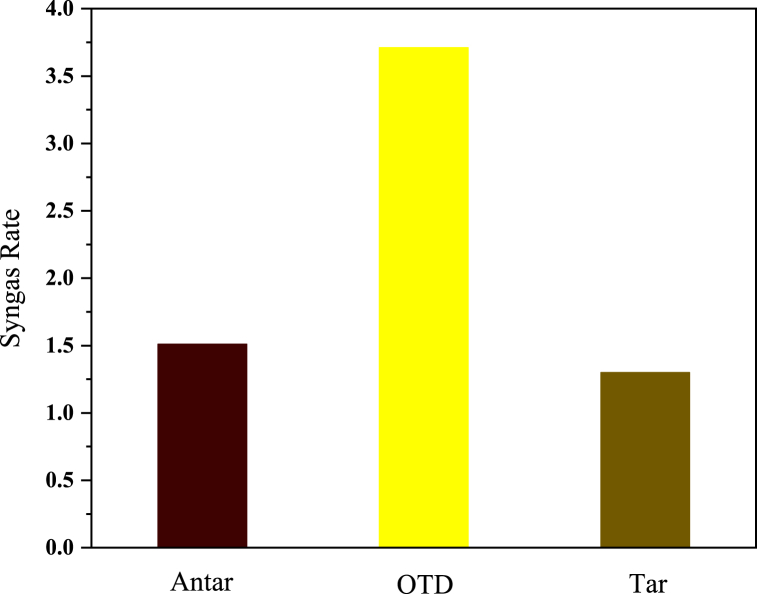
Syngas rate of Antar, OTD, and Tar waste.

As a notably important parameter, the ratio of Hydrogen to Carbon monoxide was calculated to determine whether or not the produced syngas was of high quality. Given the high efficiency of syngas, its production from wastes (hazardous) such as Antar, OTD, and Tar, which are all threats to the environment and challenges to the petrochemical industry, can be considered a great advantage. Syngas formation occurs through diverse thermochemical methodologies, such as steam reforming of hydrocarbons, gasification of biomass or coal, and partial oxidation of organic substances. Due to its adaptability as a precursor, syngas plays a crucial role in contemporary industrial operations and energy generation by serving as a versatile raw material for the synthesis of a broad spectrum of valuable chemicals and fuels [70]. The syngas ratio (H_2_/CO) in the OTD waste reaches its highest value, i.e., 3.71, followed by its values in the Antar and Tar wastes, i.e., 1.51 and 1.30, respectively. The ratio of 3.71 indicates the high Hydrogen production of syngas, which will have good efficiency when burning and produce high energy [2,3]. The gasification rate denotes how carbon-based substances convert into gaseous products, particularly hydrogen and carbon monoxide, using plasma energy. Several factors, including temperature, residence time, feedstock composition, and plasma parameters like power input and gas composition, exert influence over this rate. Optimizing the gasification efficiency, as indicated by the gasification rate, is crucial for maximizing the generation of valuable gases such as hydrogen and carbon monoxide while minimizing the formation of undesirable by-products like tars and char. Increasing gasification rates is advantageous for improving plasma pyrolysis methodologies' overall energy efficiency and economic feasibility. The gasification rate is notably impacted by the precise design of the plasma reactor, encompassing considerations such as electrode configuration and the method of plasma generation. It is crucial to optimize the reactor design to attain efficient gasification and maximize the production of desired products such as H_2_ and CO. The highest gasification rate (sum of Hydrogen and Carbon monoxide) was obtained from Tar waste, about 93.07 % (2.322SLM). In plasma pyrolysis, the combustion rate pertains to the speed at which carbon dioxide (CO_2_) and methane (CH_4_) are formed through combustion reactions occurring within the process. Although the primary emphasis in plasma pyrolysis lies in the gasification of organic substances to yield valuable hydrogen and carbon monoxide, combustion reactions may concurrently take place. The combustion rate (sum of Carbon dioxide and Methane) also changed from 5.45 % (0.055SLM) to 13.01 % (0.171SLM) in these experiments, the syngas graphs of the three wastes produced as bars in Fig. 3. As mentioned earlier, CO/CO_2_ is an important parameter, and the rate variations obtained from 8.39 % to 11.85 % in the three mentioned wastes.

Different types of Nitrogen oxides are found in nature. Nitrogen oxides are produced when fuel is burned at high temperatures, such as in combustion. The presence of NO_X_ in nature can cause some environmental repercussions, such as global warming, rhinitis, vision impairment, the formation of toxic products, and water quality degradation, to name a few. NOx emissions contribute to eutrophication, a process in which excess nitrogen compounds enter water bodies and promote the growth of algae and aquatic plants. This can lead to oxygen depletion in water, harming aquatic ecosystems and causing fish kills. NOx also plays a role in the formation of acid rain, which can damage forests, soil, and aquatic habitats [[71], [72], [73], [74], [75]]. The investigations revealed that the amount of NO_X_ gas produced during the process in the Antar waste test was only 9 ppm, which is an outstanding value. This amount equals 8 and 3 ppm in OTD and Tar waste processing, respectively.

**Fig. 4:**
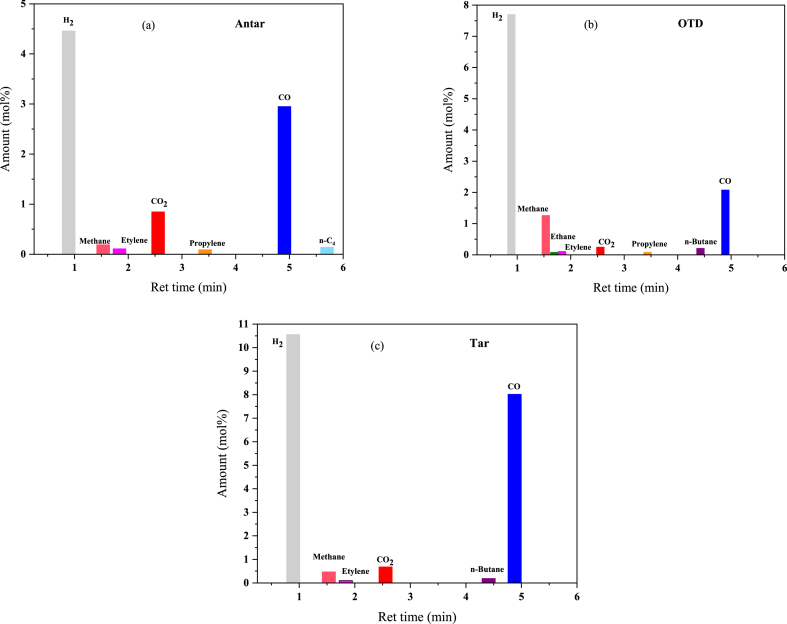
(a), (b), and (c) The gas chromatography of output products with FID and TCD.

The molar percentages of Hydrogen, Carbon monoxide, and Carbon dioxide were analyzed by the TCD detector, and those of Hydrocarbons by the FID detector. Table 6 lists the obtained percentages. The molar percentages of Hydrogen, Carbon monoxide, and Carbon dioxide in the Tar waste reach their highest values of 10.56, 8.02, and 0.68, respectively. Methane has the highest molar percentage (1.26) in OTD waste. The molar percentage values for Ethane in the Antar, Tar, and OTD wastes are 0, 0, and 0.004, respectively. The molar percentage of Ethylene in Antar and OTD wastes equals 0.11. The molar percentages of Propylene in the three Antar, OTD, and Tar wastes are 0.006, 0.003, and 0, respectively, indicating a decreasing trend. It should be noted that n-Butane did not play a role in the Antar waste and n-C4 in the OTD and Tar wastes. These results are shown in Fig. 4(a)–(c) for a better comparison.

**Table 6:** Gas chromatography of output products (mol%).

Amount (mol%)	Antar	OTD	Tar
**H**_**2**_	4.46	7.70	10.56
**CO**	2.95	2.08	8.02
**CO**_**2**_	0.25	0.25	0.68
**Methane**	0.19	1.26	0.47
**Ethane**	0	0.004	0
**Ethylene**	0.11	0.11	0.04
**Propylene**	0.006	0.003	0
**n-Butane**	0	0.21	0.19
**n-C**_**4**_	0.14	0	0

Energy density in plasma pyrolysis, expressed in kilojoules per liter (kJ/L), is a crucial parameter for evaluating the effectiveness of this high-temperature waste treatment technique. The energy density indicates the amount of energy applied per unit volume of waste, thereby determining the process's efficiency and feasibility. A high energy density guarantees the availability of adequate thermal energy to decompose waste quickly and thoroughly, thereby optimizing the conversion rate to syngas and other valuable by-products while minimizing residuals. The calculation of energy density entails dividing the total energy input into the plasma system by the volume of waste processed, highlighting the balance between energy consumption and waste throughput. Factors including the type of plasma generator, the composition of the waste, and the operational conditions (such as temperature, pressure, plasma gas flow, and etc) significantly impact the energy density. The energy density, calculated for a power of 6300 W, is 37800 kJ/L.

## 4 Conclusion

The present research employed thermal arc plasma as a novel disposal method for hazardous non-decomposable wastes, considering its beneficial characteristics, such as high-temperature generation, that considerably prevent further dangerous environmental effects. Since the transfer method enjoys high levels of stability compared to its non-transferred counterpart, it proved to be a more efficient method for waste disposal. According to our observations, the production percentage of hydrogen in the OTD waste was obtained to be about 66.24 %, which was favorable. In this regard, plasma is among the few methods yield Hydrogen at high ratios. Considering the broad applications of syngas, this research confirmed that the transferred thermal plasma torch would be an advantageous method for the disposal of industrial Tar, Antar, and OTD wastes, which, at the same time, are viable due to the production of valuable products.

## 5 Data accessibility

The datasets and code associated with this research adhere to the journal's guidelines and can be provided upon reasonable request. Data generated or analyzed during the study are available through the corresponding author. Supplementary information mentioned in the article has been incorporated into the supplementary materials for ease of access. It is important to note that this study primarily relied on pre-existing data. For further details or data requests, please contact the corresponding author at m_khani@sbu.ac.ir.

## CRediT authorship contribution statement

**Amir Hossein Kheyriyeh:** Writing – original draft, Investigation, Formal analysis, Conceptualization. **Farzaneh Ostovarpour:** Writing – review & editing, Writing – original draft, Methodology, Investigation, Formal analysis, Conceptualization. **Mohammadreza Khani:** Writing – review & editing, Writing – original draft, Visualization, Validation, Supervision, Resources, Project administration, Methodology, Investigation, Formal analysis, Data curation, Conceptualization. **Mohammad Sadegh Abbassi Shanbehbazari:** Writing – review & editing, Methodology, Investigation, Formal analysis. **Babak Shokri:** Writing – review & editing, Supervision, Project administration, Investigation, Data curation, Conceptualization.

## Declaration of competing interest

The authors declare that they have no known competing financial interests or personal relationships that could have appeared to influence the work reported in this paper.
